# Changes in Emergency Department Encounters for Vomiting After Cannabis Legalization in Colorado

**DOI:** 10.1001/jamanetworkopen.2021.25063

**Published:** 2021-09-17

**Authors:** George Sam Wang, Christine Buttorff, Asa Wilks, Daniel Schwam, Gregory Tung, Rosalie Liccardo Pacula

**Affiliations:** 1 Department of Pediatrics, University of Colorado Anschutz Medical Campus, Children's Hospital Colorado, Aurora; 2 RAND Corporation, Arlington, Virginia; 3 RAND Corporation, Santa Monica, California; 4 Department of Health Systems, Management & Policy, Colorado School of Public Health, University of Colorado Anschutz Medical Campus; 5 Sol Price School of Public Policy, Schaeffer Center for Health Policy & Economics, University of Southern California, Los Angeles

## Abstract

**Importance:**

Cannabis hyperemesis syndrome is an emerging clinical issue associated with cannabis use. Legalization of cannabis has led to an increase in vomiting-related illnesses in health care settings.

**Objective:**

To examine whether legalization of cannabis in Colorado has been associated with increases in vomiting-related emergency department (ED) visits.

**Design, Setting, and Participants:**

A cross-sectional design was used to assess the increase in ED claims for vomiting reported to the Colorado Hospital Association between January 1, 2013, and December 31, 2018, in counties that had no prior cannabis dispensaries before legalization compared with those that did. A total of 820 778 patients seeking care through Colorado EDs were included in the analysis.

**Exposures:**

The number of medical and recreational cannabis dispensaries per county per quarter.

**Main Outcomes and Measures:**

County per capita rate of vomiting-related ED claims per quarter.

**Results:**

Vomiting-related ED health care encounters increased from 119 312 in 2013 to 153 699 in 2018 (29% increase). Over this period, 203 861 patients (25%) were aged 0 to 18 years; 114 201 (14%) were aged 19 to 25 years, and 502 771 (61%) were aged 26 years or older; 510 584 patients (62%) were female. Additional recreational dispensaries were associated with increased vomiting-related ED visits (incidence rate ratio, 1.03; 95% CI, 1.01-1.05), but counties with high baseline medical dispensary exposure experienced smaller increases in vomiting-related ED visits than counties with no baseline medical dispensary exposure (incidence rate ratio, 0.97; 95% CI, 0.95-0.99). Counties with a high number of medical marijuana dispensaries had increases at a 5.8% slower rate than counties with none.

**Conclusions and Relevance:**

The findings of this study suggest that cannabis legalization in Colorado is associated with an increase in annual vomiting-related health care encounters with regard to exposure to these markets. It may be useful for health care clinicians to be aware of cannabis hyperemesis syndrome and inquire about cannabis use when appropriate.

## Key Points

**Question:**

Has the number of vomiting-related emergency department visits increased after recreational cannabis legalization in Colorado?

**Findings:**

In this cross-sectional study of 820 778 patients seeking care through Colorado emergency departments, cannabis legalization was associated with an increase in annual vomiting-related health care encounters. The highest increases were observed in counties without existing medical dispensaries.

**Meaning:**

These findings suggest that health care clinicians in states that have legalized cannabis should be aware of symptoms associated with cannabis hyperemesis syndrome; documentation may help ensure accurate public health surveillance on consequences associated with cannabis legalization.

## Teaser

This cross-sectional study examines changes in the rates of emergency department presentation by individuals with cannabis-related vomiting since legalization of cannabis in Colorado.

## Introduction

Cannabis can have antiemetic properties; a synthetic cannabinoid derivative has been approved by the US Food and Drug Administration for treatment of chemotherapy-induced nausea and vomiting.^1^ Paradoxically, an increasing health concern is associated with chronic cannabis use: cannabis hyperemesis syndrome (CHS).^2^ Although there are no definitive criteria that exist for CHS, this clinical syndrome consists of frequent episodes of recurrent nausea, vomiting, and severe abdominal pain that is often relieved by compulsive hot bathing.^3,4,5,6,7,8^ The frequency of the vomiting is similar to other cyclic vomiting conditions. Although not entirely clear, it is hypothesized that the cyclic vomiting is due to a disequilibrium with the endocannabinoid system and the brainstem and hypothalamic-pituitary-adrenal axis after heavy cannabis use.^8^ The duration, frequency, route, or potency of cannabis products used that leads to greater risk for developing this illness has not been determined. However, a review of more than 200 patients diagnosed with CHS revealed that daily use of cannabis was reported in 47.9% of the patients and weekly use in 19.4% of the patients.^9^ Often, patients with CHS can compile significant health care expenses due to frequent emergency department (ED) visits and extensive medical and surgical evaluations.^10,11^ In addition, symptoms of CHS can be refractory to conventional treatments and symptomatic care can require hospital admission.^7,9,11,12,13,14^

As of June 1, 2021, more than half of US states have allowed cannabis for medical use, and 16 states have allowed recreational sales. Surveillance research has reported that legalization has been associated with an overall increase in adult and young adult cannabis use as well as overall reported frequency in use and use of higher-potency products.^15,16,17^ There is substantial concern that this increase in long-term and frequent cannabis use, especially with products containing high amounts of tetrahydrocannabinol (THC),^18^ will lead to an increase in the prevalence of CHS. Two urban academic EDs in Colorado, a state that has legalized use of medical and recreational cannabis, noted a doubling of cyclic vomiting presentations after legalization of medical cannabis, and gastrointestinal illness was a common reason for seeking care after cannabis use.^19,20^ Another study examining inpatient visits across Colorado before and after legalization of recreational cannabis found a 46% increase in statewide hospitalizations for cyclical vomiting in just 5 years.^21^ Nationally, hospitalization rates for cyclical vomiting syndrome have increased by 60% between 2005 and 2014, with cannabis involvement increasing during this time from 2.2% of these cases in 2005 to 21.2% in 2015.^22^ Although suggestive of an association, the determination of an association between legalization policies and the incidence of CHS has not been established.

The objective of this study was to evaluate the association between cannabis legalization and vomiting-related health care encounters in Colorado to estimate whether the opening of new markets may generate an increase in these cases. Because counties with greater numbers of medical dispensaries would have greater baseline exposure to cannabis, we hypothesized that the increase in the number of vomiting-related ED visits after legalization of recreational cannabis might be higher in counties with low or no baseline exposure to medical markets before legalization.

## Methods

We used a cross-sectional study design to make use of the natural variation that occurs in exposure to recreational cannabis dispensaries across Colorado after the state granted jurisdictions their own authority to permit or ban recreational outlets after legalization. Information from 2 statewide administrative data sources covering the study period of 2013 to 2018 was combined to conduct this analysis. First, cannabis dispensary data were obtained from the Colorado Department of Revenue Marijuana Enforcement Division, which manages the licensing of medical and recreational dispensary licenses for the state. The Department of Revenue provided dispensary information (name and address including zip code) and date of licensure for all medical and recreational dispensaries. Second, data on all ED claims involving vomiting reported to the Colorado Hospital Association (CHA) were linked to the location of dispensaries using the patient’s zip code. The RAND Human Subjects Protection Committee exempted this research from human participant protection because it did not contain any identifying personal patient information from existing data sources. This study followed the Strengthening the Reporting of Observational Studies in Epidemiology (STROBE) reporting guideline.

Owing to a lack of a primary *International Classification of Diseases* (*ICD*) code and concern for underdiagnosis and recognition, vomiting-involved cases were identified using *International Classification of Diseases, Ninth Revision* (*ICD-9*) (codes: 536.2 and 787*) and *International Statistical Classification of Diseases and Related Health Problems, 10th Revision* (*ICD-10*) (codes: R11.1 and G43.A) codes listed for any diagnostic code for vomiting. Vomiting cases co-coded with cannabis *ICD* codes (*ICD-9* codes: 304.3 and 305.2; *ICD-10* code: F12*) were also obtained. Most hospitals and health care systems (>100) in Colorado contribute health care encounter data to the CHA, although not consistently every month. Emergency department visits, however, did not start being reported statewide until 2013. Emergency department visits that resulted in hospitalization were not included in the analysis. Additional variables collected from the CHA included demographic characteristics (age and sex), patient’s residence zip code, and insurance type. The data were aggregated to the county level per quarter based on patient residence and then matched to the licensing data. We did not have a unique person identifier that allowed us to track multiple visits for the same person.

Patient exposure to cannabis markets was determined by 2 indicators: baseline exposure to medical dispensaries and then growth in new recreational stores after legalization. Both of these measures were examined in absolute terms (ie, the number of licensed medical or recreational dispensaries within the county) as well as a relative term (the number of licensed medical or recreational dispensaries per capita). As noted previously, local jurisdictions in Colorado were given authority to decide whether and when to allow medical or recreational dispensaries, creating local variation in exposure to cannabis markets over time. Medical cannabis was legalized in 2000 in Colorado, but the significant increase in the number of patients using medical cannabis did not occur until near the end of 2009.^23^ Colorado did not require licensing of medical cannabis dispensaries until 2010, at which point there was substantial growth until the end of 2012 when Colorado legalized recreational cannabis. Recreational cannabis sales did not begin until January 2014 and were initially limited to a subset of existing medical cannabis dispensaries until September 2014, when independent recreational cannabis dispensaries began operating.

We categorized counties in terms of the relative size of the medical cannabis market before recreational cannabis legalization (in the third quarter of 2012) as follows: no market (0 licensed medical dispensaries), low market (1-9 licensed medical dispensaries), and high market (≥10 licensed medical dispensaries). These categories were based on the mean (SD) number of 7.86 (28.01) medical dispensaries in 2012, quarter 3, licensed medical dispensaries. The number of licensed recreational dispensaries was then used as our main exposure variable, interacted with the medical dispensary categorical measure.

We used descriptive analyses to examine vomiting-related ED visits over time and a high-dimensional fixed-effects Poisson regression model that allowed us to account for the nonnegative nature of the data, the presence of a large number of counties that had 0 outcomes during our sample period, and the desire to control for unobserved fixed heterogeneity across counties through fixed effects.^24,25^ We used the *ppmlhdfe* command in Stata, version 16 (StataCorp LLC), adjusting for population exposure for all our multivariate analyses (we use the census county-level annual population data).^26^ The full regression results are in the eTables 2 to 4 in the Supplement.

We conducted several additional sensitivity analyses (eTable 2 and eTable 3 in the Supplement): (1) a version using a negative binomial model (run with Stata’s *ngbreg* command) to account for overdispersion, (2) recoding the number of recreational dispensaries into a categorical variable based on the quantiles of the distribution in the fourth quarter of 2016 when the number of recreational dispensaries per county stabilized, and (3) 2 models to assess the association between Medicaid expansion as part of the Affordable Care Act in 2014 and the number of ED visits separate from the outcome associated with recreational dispensaries–the number of vomiting-related ED visits for commercial enrollees, which should be unaffected by the Medicaid expansion and the number of Medicaid enrollees, which should be unrelated to recreational sales.

## Results

From January 1, 2013, through December 31, 2018, there were a total of 820 778 vomiting-related ED visits. Annual vomiting-related health care encounters increased from 119 312 in 2013 to 153 699 by 2018 (a 29% increase). The demographic characteristics of the patients represented in these claims data were as follows: 203 861 patients (25%) were aged 0 to 18 years; 114 201 (14%) were aged 19 to 25 years, and 502 771 (61%) were aged 26 years and older. Most patients (510 584 [62%]) were female; 310 005 (38%%) were male. The most common insurance type was government/public (505 002 [62%]), followed by commercial (235 302 [29%]), uninsured (63 257 [8%]), other (12 442 [2%]), unknown (2232 [0.3%]), and workers’ compensation (1598 [0.2%]).

**Figure 1.**
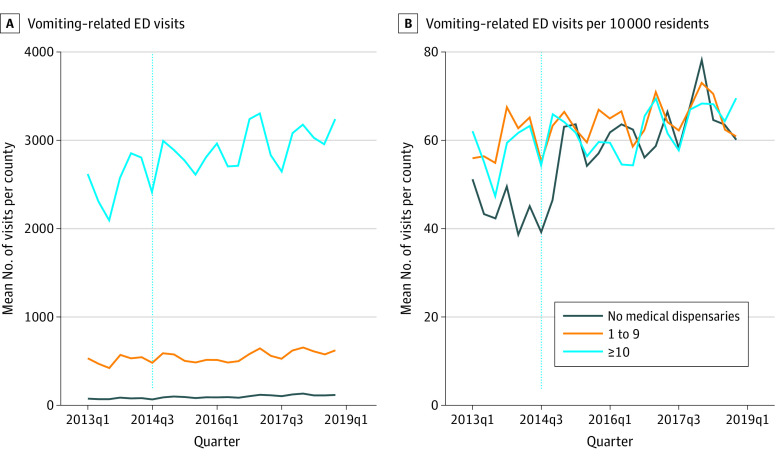
Changes in Vomiting-Related Emergency Department (ED) Visits Over Time, by Baseline Medical Dispensary Exposure Mean number of quarterly vomiting-related ED visits per county (A) and per 10 000 residents per county (B).

In counties with no medical dispensaries, vomiting-related ED visits increased from a mean of 76.94 (51.24 per 10 000) in 2013, quarter 1, to 115.22 (60.16 per 10 000) in 2018, quarter 4 (Figure 1). In comparison, counties with low medical dispensaries increased from 534.42 (55.97 per 10 000) to 621.81 (60.85 per 10 000) and counties with high medical dispensaries increased from 2619.50 (62.09 per 10 000) to 3248.83 (69.55 per 10 000) over the same period. Because counties with no or low numbers of medical dispensaries also tend to have lower population levels, the number of vomiting-related ED visits was lower (Figure 1A) in these counties compared with those with high baseline numbers of medical dispensaries. After standardizing by population, counties with no baseline exposure had greater increases in vomiting-related ED visits after recreational cannabis legalization and allowance for new recreational dispensaries (September 2014) than counties with low or high baseline medical cannabis dispensaries (Figure 1B). The number of cases for which a cannabis *ICD* code was also included as a diagnosis for the ED visit increased from 0.3% in 2013 to 2.3% at the end of 2018. Although this change represents over a 7-fold increase from baseline, the overall rate of co-occurrence remained low.

**Figure 2.**
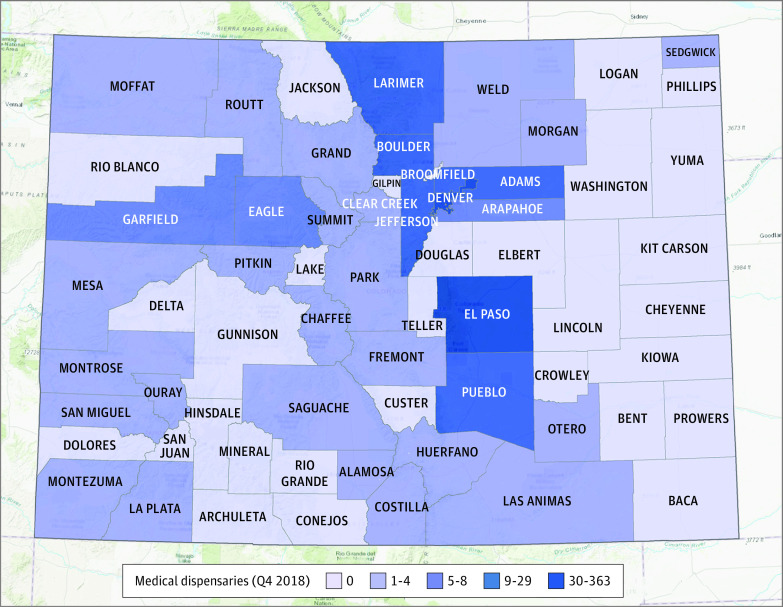
Variation in Number of Recreational Dispensaries by County in 2018 Map generated by OpenStreetMap.^27^

Table 1 provides additional descriptive statistics on our main outcome and exposure measures. Over the study period, the mean (SD) number of county-level vomiting-related ED visits increased from 466.1 (950.3) in 2013 to 600.3 (1260.4) in 2018—a 28.8% increase over the period. Mean (SD) per capita rates per county increased as well, from 52.4 (39.4) per 10 000 residents in 2013 to 66.8 (42.1) per 10 000 residents in 2018. At the same time, the mean number of medical cannabis dispensaries per county held steady, at approximately 8 during the study period, while the mean number of recreational dispensaries per county per quarter increased from 3.6 (14.8) in 2014 to 8.5 (23.5) in 2018. Figure 2 shows the variation in the number of recreational dispensaries across Colorado counties.^27^ In 2018, quarter 4, there were 1037 dispensaries statewide: 552 recreational dispensaries and 485 medical dispensaries. The highest total dispensary counts were found in urban counties: Denver (357), El Paso (136), Boulder (82), Pueblo (52), and Jefferson (49) county.

**Table 1.** Descriptive Statistics, County-Level Exposure, and Outcome Measures by Year

Variable	Mean (SD)						
	Full period (2013-2018)	2013	2014	2015	2016	2017	2018
County-level vomiting-related ED visits							
No. of vomiting visits	534.4 (1123.6)	466.1 (950.3)	518.2 (1103.3)	517.1 (1073.8)	529.8 (1136.3)	574.9 (1198.3)	600.3 (1260.4)
Vomiting visits per 10 000 residents	59.7 (38.8)	52.4 (39.4)	52.0 (36.0)	61.3 (37.8)	61.6 (37.0)	64.3 (37.3)	66.8 (42.1)
County-level measures of cannabis market							
No. of medical dispensaries	8.1 (28.6)	8.1 (28.3)	7.9 (27.2)	8.1 (28.2)	8.3 (30.1)	8.1 (29.4)	7.8 (28.5)
Medical dispensaries per 10 000 residents	0.9 (1.5)	1.1 (1.8)	1.0 (1.6)	1.0 (1.5)	0.9 (1.3)	0.9 (1.3)	0.8 (1.1)
No. of recreational dispensaries	5.5 (18.5)	0	3.6 (14.8)	6.0 (18.4)	7.0 (20.3)	7.8 (22.3)	8.5 (23.5)
Recreational dispensaries per 10 000 residents	1.7 (3.9)	0	0.8 (1.7)	1.9 (3.9)	2.3 (4.4)	2.4 (4.6)	2.6 (5.1)
No. of total dispensaries	13.5 (44.0)	8.1 (28.3)	11.5 (40.9)	14.1 (45.1)	15.3 (48.0)	15.9 (49.0)	16.3 (49.0)

Abbreviation: ED indicates emergency department.

Overall, additional recreational dispensaries in a county were associated with an increase in vomiting-related ED visits when measured in terms of absolute numbers (incidence rate ratio, 1.03; 95% CI, 1.01-1.05) (Table 2). Counties with high baseline medical dispensary exposure experienced fewer vomiting-related ED visits than counties with no baseline exposure (incidence rate ratio, 0.97; 95% CI, 0.95-0.99). We found consistent results when per capita exposure measures were used instead of count measures, although an increase in per capita visits in counties with few medical marijuana dispensaries at baseline increased at a rate similar to counties with no medical marijuana dispensaries. Counties with high baseline exposure to medical marijuana dispensaries increased at a 5.8% slower pace than counties with no exposure, based on combined incidence rate ratios (Table 2). The full results in eTable 1 in the Supplement show the increasing association between year and the outcome, which may reflect Medicaid expansion.

**Table 2.** High Dimensional Fixed-Effect Regression Results for Association Between Exposure and County Quarterly Vomiting-Related ED Visits^a^

Outcome: quarterly county-level vomiting-related ED visits per 10 000 residents	IRR (95% CI)	
	Count exposure	Per capita exposure
Recreational dispensary count	1.03 (1.01-1.05)^b^	NA
Baseline medical (low) recreational dispensary count^c^	0.97 (0.95-0.98)^d^	NA
Baseline medical (high) recreational dispensary count^c^	0.97 (0.95-0.99)^b^	NA
Recreational dispensary count per 10 000 residents	NA	1.04 (1.02-1.06)^d^
Baseline medical (low) recreational dispensary count per 10 000 residents^c^	NA	0.95 (0.89-1.01)
Baseline medical (high) recreational dispensary count per 10 000 residents^c^	NA	0.90 (0.85-0.96)^b^
Post *ICD-9* indicator	0.91 (0.85-0.97)^b^	0.91 (0.85-0.97)^b^
County unemployment rate	0.98 (0.90-1.07)	1.00 (0.91-1.09)
Total hospital admissions	1.00 (1.00-1.00)	1.00 (1.00-1.00)
Combined IRR for increase in recreational dispensaries		
No medical dispensary exposure	1.030	1.042
*P* value	.002	<.001
Low medical dispensary exposure	0.995	0.987
*P* value	.11	.73
High medical dispensary exposure	0.999	0.942
*P* value	.02	.06

Abbreviations: ED indicates emergency department; *ICD-9*, *International Classification of Diseases, Ninth Revision*; IRR, incidence rate ratio; NA, not applicable.

^a^ Counties were categorized according to the number of medical dispensaries in the county at baseline (third-quarter 2012) before legalization of recreational cannabis (no, 0 medical dispensaries; low, 1-9; high, ≥10). All models included quarter, year, and county fixed effects; these results are not included in the Table. All models were estimated using *ppmlhdfe* in Stata, version 16, with county population as the exposure variable.

^b^ Significant at the 1% level.

^c^ Significant at the 5% level.

^d^ Significant at the 0.01% level.

We conducted several sensitivity analyses for our main findings. The results based on the number of vomiting-related ED visits for patients with commercial insurance were similar to the main results (eTable 2 in the Supplement), where counties with no baseline medical dispensaries had higher rates of vomiting-related ED visits than counties with low or high levels of baseline medical dispensaries. Regarding the number of Medicaid enrollees, the results suggest there may be an association between counties with higher recreational dispensaries and Medicaid enrollment. However, it is unlikely to be a factor in the main results because we observed similar findings for the commercially insured population, and the direct effects on Medicaid enrollment were small compared with the findings observed for the main results. Results of using the negative binomial model were similar (eTable 3 in the Supplement). When using the number of dispensaries as a categorical variable, the changes were in a similar direction as the main result, although higher in magnitude, and the significance did not change (eTable 4 in the Supplement).

## Discussion

Our analysis suggests that the number of vomiting-related health care encounters in Colorado has significantly increased in all counties since recreational cannabis was legalized in 2014, with a nearly 30% increase in annual cases overall. In addition, the data indicate that, although counties with high baseline medical dispensary exposure saw large increases in terms of raw counts, counties with no previous baseline exposure experienced larger increases in both percentage and population-adjusted rates. As depicted in Figure 1, the rates of county-level ED visits related to vomiting between no-exposure counties and low- or high-exposure counties basically were no longer different after recreational dispensaries were allowed to open, despite there being some fairly large differences in the quarterly per capita rates across these counties before new recreational dispensaries opened.

These results suggest counties with no baseline exposure to medical cannabis markets experienced more vomiting-related health events with the new opening of recreational cannabis dispensaries than counties already introduced to these markets through medical dispensaries. This difference may be due to a lack of medical oversight regarding use in counties without a preexisting or coexisting medical market. Recreational cannabis does not require a physician recommendation, and any adult can purchase recreational cannabis at a dispensary. The difference may also be associated with the increased availability of high-potency cannabis products that became available with the introduction of the recreational market, which would explain why all counties experienced an increase in vomiting-related ED visits after recreational cannabis legalization.

These results also suggest that cannabis comorbidity is likely being underidentified in these vomiting cases. Although there was a 7-fold increase in the *ICD* coding of cannabis use with vomiting-related illness for health care encounters in Colorado, there were still too few to have a sufficiently powered analysis to examine them directly. Other research using the National Inpatient Sample found a 10-fold increase in cyclical vomiting hospitalization with documented concurrent cannabis use, increasing from 2.2% in 2005 to 21.2% in 2014 nationally.^22^ The low rate of cannabis involvement in ED visits in Colorado is surprising in light of this evidence and the fact that Colorado has a higher annual prevalence rate of cannabis use and cannabis use order than the nation overall.^28^

Proper identification of cannabis-related vomiting symptoms is important to prevent complications and provide counseling on cannabis cessation; intense, protracted vomiting has been associated with severe and fatal outcomes.^29^ It may be useful to train clinicians and encourage them to document cannabis use in relation to vomiting-related health care encounters. Although accurate identification of CHS continues to be problematic because the pathophysiologic factors of CHS are not fully understood, greater effort in identification of the presence of likely confounders is necessary. It has been proposed that these symptoms stem from chronic and frequent cannabis use, which leads to an imbalance of the endocannabinoid system and the hypothalamic-pituitary-axis, similar to other cyclic vomiting syndromes.^8,9^ Although low-level THC has antiemetic properties, it is postulated that higher amounts of THC lead to a paradoxical proemetic/nausea state.^30,31^ The proposed dose response is of concern because cannabis legalization and introduction of a mature cannabis commercial market to areas with no experience to medical markets provides a venue to purchase high-potency cannabis products. Concentrated products, such as THC-infused food products (edibles) and concentrates (waxes, budders, and oils) can contain substantial and high amounts of THC.^32^

### Limitations

This study has limitations. First, we were only able to identify the number of licensed dispensaries operating in each county and were unable to account for unlicensed dispensaries. Our inability to capture these unlicensed dispensaries would be a problem for our identification strategy only if the unlicensed dispensaries were more likely to exist in counties with no licensed dispensaries before legalization. Although possible, the inability to identify unlicensed dispensaries would imply that our estimate of the association between legal cannabis markets and harm is biased toward a decrease, and the true effect is likely larger. Second, although CHA represents nearly all Coloradans with an ED visit, there is variation in the hospitals reporting data to CHA each year. Furthermore, hospitals are more narrowly geographically located than other medical care services—45 of Colorado’s 64 counties have a hospital, 57% of which are in urban areas. There also was some ecological fallacy. There is no perfect administrative data source that contains both individual-level health outcomes and individual-level marijuana use at the substate level. Survey data sets contain this information but generally are not powered for substate analyses. We tried to reduce the influence of these concerns by using the patient’s place of residence for mapping county visit to available dispensaries rather than the hospital’s county. In addition, the ED data provided to us from CHA excluded patients who were admitted to the hospital. Hence, our analysis may underestimate the total association between the cannabis market opening and the need for ED services.

## Conclusions

The findings of this study suggest a substantial increase in vomiting-related ED visits in Colorado after legalization of recreational cannabis. In Colorado, there was an overall 29% increase in vomiting-related health care encounters since the opening of recreational cannabis markets. The increase was greatest in counties where there was no prior medical cannabis market, although data were insufficient to directly evaluate cannabis-involved cyclical vomiting episodes. It would be useful for health care clinicians in states that have legalized cannabis and allow for commercial markets to know the constellation of CHS symptoms, inquire about cannabis use when appropriate, and be aware of the available treatment options. Not doing so may impede physicians’ ability to meet the needs of many patients requiring emergency services and make it difficult to accurately monitor the public health association of cannabis use and legalization with a range of health measures, including cardiovascular health, pulmonary disease, injury, addiction, and behavioral health.^33^

## Notes

Supplement. **eTable 1.** Descriptive Statistics, County-level Exposure, and Outcome Measures by Year **eTable 2.** Sensitivity Analysis for Visits With Commercial Insurance and Medicaid Enrollees **eTable 3.** Sensitivity Analysis, Negative Binomial Regression Results **eTable 4.** Vomiting-related ED Visits, Using the Number of Recreational Dispensaries as a Categorical Variable
