# Supplementary material for: Changes in Emergency Department Encounters for Vomiting After Cannabis Legalization in Colorado
Source: JAMA Netw Open. 2021 Sep 17;4(9):e2125063. doi: 10.1001/jamanetworkopen.2021.25063 (PMC8449280; doi:10.1001/jamanetworkopen.2021.25063)
Supplement: Supplement. — eTable 1. Descriptive Statistics, County-level Exposure, and Outcome Measures by Year eTable 2. Sensitivity Analysis for Visits With Commercial Insurance and Medicaid Enrollees eTable 3. Sensitivity Analysis, Negative Binomial Regression Results eTable 4. Vomiting-related ED Visits, Using the Number of Recreational Dispensaries as a Categorical Variable [file jamanetwopen-e2125063-s001.pdf]

Supplementary Online Content

Wang GS, Buttorff C, Wilks A, Schwam D, Tung G, Pacula RL. Changes in emergency department encounters for vomiting after cannabis legalization in Colorado. *JAMA Netw Open*. 2021;4(9):e2125063. doi:10.1001/jamanetworkopen.2021.25063

eTable 1. Descriptive Statistics, County-level Exposure, and Outcome Measures by Year

eTable 2. Sensitivity Analysis for Visits With Commercial Insurance and Medicaid Enrollees

eTable 3. Sensitivity Analysis, Negative Binomial Regression Results

eTable 4. Vomiting-Related ED Visits, Using the Number of Recreational Dispensaries as a Categorical Variable

This supplementary material has been provided by the authors to give readers additional information about their work.

© 2021 Wang GS et al. *JAMA Network Open.*

eTable 1: Descriptive Statistics, County-level Exposure and Outcome Measures, by Year

		Full Period (2013-2018)	2013	2014	2015	2016	2017	2018
	**County-level Vomiting Related ED Visits, Mean (SD)**							
Number of Vomiting Visits		534.4 (1123.6)	466.1 (950.3)	518.2 (1103.3)	517.1 (1073.8)	529.8 (1136.3)	574.9 (1198.3)	600.3 (1260.4)
Vomiting Visits per 10K		59.7 (38.8)	52.4 (39.4)	52.0 (36.0)	61.3 (37.8)	61.6 (37.0)	64.3 (37.3)	66.8 (42.1)
	**County-level Measures of Cannabis Market, Mean (SD)**							
Number of Medical Dispensaries		8.1 (28.6)	8.1 (28.3)	7.9 (27.2)	8.1 (28.2)	8.3 (30.1)	8.1 (29.4)	7.8 (28.5)
Medical Dispensaries per 10k		0.9 (1.5)	1.1 (1.8)	1.0 (1.6)	1.0 (1.5)	0.9 (1.3)	0.9 (1.3)	0.8 (1.1)
Number of Recreational Dispensaries		5.5 (18.5)	0.0 (0.0)	3.6 (14.8)	6.0 (18.4)	7.0 (20.3)	7.8 (22.3)	8.5 (23.5)
Rec Dispensaries per 10k		1.7 (3.9)	0.0 (0.0)	0.8 (1.7)	1.9 (3.9)	2.3 (4.4)	2.4 (4.6)	2.6 (5.1)
Number of Total Dispensaries		13.5 (44.0)	8.1 (28.3)	11.5 (40.9)	14.1 (45.1)	15.3 (48.0)	15.9 (49.0)	16.3 (49.0)

SD: Standard Deviation

© 2021 Wang GS et al. *JAMA Network Open.*

eTable 2: Sensitivity Analysis for Visits with Commercial Insurance and Medicaid Enrollees

Vomiting-Related ED Visits	Commercial Vomiting-Related ED Visits		Number of Medicaid Enrollees	
	Count Exposure	Per Capita Exposure	Count Exposure	Per Capita Exposure
	Coeff [95% CI]	Coeff[95% CI]	Coeff [95% CI]	Coeff [95% CI]
Rec Dispensary Count	0.03 [0.01,0.04]***		0.002 [0.00,0.00]*	
Baseline Medical (low) * Rec Dispensary Count	-0.03 [-0.05,-0.02]***		-0.002 [-0.00,-0.00]*	
Baseline Medical (high) * Rec Dispensary Count	-0.029 [-0.04,-0.01]***		-0.002 [-0.00,-0.00]*	
Rec Dispensary Count per 10k population		0.04 [0.02,0.06]***		0.002 [0.00,0.00]**
Baseline Medical (low) * Rec Dispensary Count per 10k		-0.05 [-0.13,0.03]		-0.001 [-0.01,0.00]
Baseline Medical (high) * Rec Dispensary Count per 10k		-0.08 [-0.15,-0.01]*		-0.005 [-0.02,0.01]
Seasonal Quarter (Q1 Apr-June Reference Group)				
Second Q (Apr-June)	-0.08 [-0.09,-0.06] ***	-0.08 [-0.09,-0.06]***	0.00 [-0.00,0.00]	0.00 [-0.00,0.00]
Third Q (July-Sept)	0.13 [-0.14,-0.12] ***	-0.13 [-0.15,-0.12]***	0.00 [-0.00,0.00]	0.00 [-0.00,0.00]
Fourth Q (Oct-Dec)	0.03 [0.01,0.05]**	0.03 [0.00,0.05]*	0.00 [-0.00,0.00]	0.00 [-0.00,0.00]
Year (2013 Reference Group)				
2014	0.37 [0.20,0.54]***	0.36 [0.18,0.55]***	-0.03 [-0.05,-0.00]*	-0.03 [-0.05,-0.01]**
2015	0.48 [0.21,0.75]***	0.48 [0.19,0.76]**	-0.05 [-0.08,-0.02]**	-0.05 [-0.09,-0.02]***
2016	0.61 [0.31,0.91]***	0.61 [0.28,0.93]***	-0.07 [-0.11,-0.03]***	-0.07 [-0.11,-0.04]***
2017	0.73 [0.36,1.10]***	0.72 [0.33,1.11]***	-0.09 [-0.14,-0.04]***	-0.09 [-0.14,-0.05]***
2018	0.73 [0.39,1.07]***	0.71 [0.36,1.06]***	-0.09 [-0.15,-0.05]***	-0.10 [-0.15,--0.06]***

© 2021 Wang GS et al. *JAMA Network Open.*

Post ICD9 Indicator	-0.09 [-0.16,-0.03]**	-0.09 [-0.16,-0.03]**	0.00 [-0.00,0.00]	0.00 [-0.00,0.00]
Unemployment Rate	0.02 [-0.07,0.11]	0.02 [-0.07,0.12]	-0.01 [-0.02,0.00]	-.01 [-0.02,0.00]
Total Hospital Admissions per capita	0.00 [-0.00,0.00]	0.00 [-0.00,0.00]	0.00 [-0.00,0.00]	0.00 [-0.00,0.00]
Constant	-5.59 [-6.36,-4.84]***	-5.79 [-6.54,-5.06]***	-1.62 [-1.69,-1.54]***	-1.62 [-1.69,-1.55]***
N	1536	1536	1536	1536

CI: Confidence Interval. Note: Counties were categorized according to the number of medical dispensaries in the county at baseline (third quarter 2012) prior to the legalization of recreational cannabis (no = zero medical dispensaries; low = 1-9; high = 10+). *** Indicates significance at the 0.01% level, ** significance at the 1% level, * significance at the 5% level. All models estimated using ppmlhdfe in Stata 16.1 with county population as our exposure variable. We present coefficients, rather than incidence rate ratios (IRRs) for this table due to the very small magnitude IRRs.

The Medicaid expansion, enacted as part of the Affordable Care Act, occurred at the same time as the start of recreational cannabis sales in Colorado in 2014. The purpose of this sensitivity analysis was to check whether the Medicaid expansion in 2014 could be the associated with additional ED visits unrelated to cannabis use. We assessed the number of vomiting-related ED visits for commercial enrollees, which should be unaffected by the ACA (largely because the number of marketplace enrollees in Colorado was very small in 2014 – approximately 125,000 people out of approximately 5 million). Additionally, we examined the number of Medicaid enrollees as a falsification test, which should be unrelated to recreational sales.

The commercial payer results are similar to the main results (eTable 1), where we find counties with no baseline medical dispensaries have higher rates of vomiting ED visits than those counties with low or high levels of baseline medical exposure. On the right panel, we examined the number of Medicaid enrollees as the outcome, and find that while these variables are statistically significant at the 5% level, none are large in magnitude. Given the small size of the coefficients, especially in relation to the commercial, results, we do not think the Medicaid expansion is driving the result. Additionally, the correlation between Medicaid enrollment and recreational dispensaries appears to be consistent across counties with differential medical marijuana exposure at baseline, and so not driving any of our results from the main specification.

© 2021 Wang GS et al. *JAMA Network Open.*

eTable 3: Sensitivity Analysis, Negative Binomial Regression Results

Vomiting-Related ED Visits	Count Exposure	Per Capita Exposure
	IRR [95% CI]	IRR [95% CI]
Rec Dispensary Count	1.02 [1.00,1.04]+	
Baseline Medical (low) * Rec Dispensary Count	0.96 [0.94,0.99]**	
Baseline Medical (high) * Rec Dispensary Count	0.98 [0.96,1.00]*	
Rec Dispensary Count per 10k population		1.04 [1.01,1.08]*
Baseline Medical (low) * Rec Dispensary Count per 10k		0.91 [0.82,1.01]+
Baseline Medical (high) * Rec Dispensary Count per 10k		0.81 [0.71,0.92]**
Baseline Medical (low)	1.14 [0.85,1.53]	1.42 [0.94,2.15]+
Baseline Medical (high)	0.92 [0.70,1.21]	1.51 [1.02,2.25]*
Seasonal Quarter (Q1 Jan-Mar Reference Group)		
Second Q (Apr-June)	0.98 [0.95,1.01]	0.93 [0.91,0.94]***
Third Q (July-Sept)	0.91 [0.88,0.93]***	0.87b[0.86,0.88]***
Fourth Q (Oct-Dec)	1.00 [0.96,1.04]	1.03b[1.01,1.06]**
Year (2013 Reference Group)		
2014	1.02 [0.93,1.13]	1.16 [1.04,1.29]**
2015	1.23 [1.06,1.44]**	1.19 [1.04,1.35]**
2016	1.28 [1.07,1.54]**	1.30 [1.12,1.50]***
2017	1.38 [1.14,1.67]***	1.39 [1.24,1.56]***
2018	1.44 [1.17,1.77]***	1.45 [1.28,1.63]***
Post ICD9 Indicator	0.97 [0.86,1.08]	0.91 [0.85,0.97]**
Total Hospital Admissions	1.00 [1.00,1.00]**	1.00 [1.00,1.00]
Constant	0.00 [0.00,0.01]***	0.00 [0.00,0.01]***
Ln alpha	0.31 [0.24,0.40]***	
N	1536	1536

Note: Counties were categorized according to the number of medical dispensaries in the county at baseline (third quarter 2012) prior to the legalization of recreational cannabis (no = zero medical dispensaries; low = 1-9; high = 10+). *** Indicates significance at the 0.01% level, ** significance at the 1% level, * significance at the 5% level, and + indicates significance at 10% level. These models were run with nbreg in State 16.1. County fixed effects were not used in this model.

© 2021 Wang GS et al. *JAMA Network Open.*

eTable 4. Vomiting-Related ED Visits, Using the Number of Recreational Dispensaries as a Categorical Variable

	IRR [95% CI]
Recreational Dispensary Category 2 (1, reference)	1.15 [0.95,1.40]
Recreational Dispensary Category 3	1.44 [1.04,1.99]*
Recreational Dispensary Category 4	2.36 [0.78,7.11]
Baseline Medical (low) * Rec. Dispensary Category 2	0.74 [0.39,1.39]
Baseline Medical (low) * Rec. Dispensary Category 3	0.67 [0.48,0.92]*
Baseline Medical (low) * Rec. Dispensary Category 4	0.39 [0.13,1.19]+
Baseline Medical (high) * Rec. Dispensary Category 2	0.94 [0.78,1.13]
Baseline Medical (high) * Rec. Dispensary Category 3	0.81 [0.59,1.12]
Baseline Medical (high) * Rec. Dispensary Category 4	0.38 [0.12,1.14]+
Seasonal Quarter (Q1 Jan-Mar Reference Group)	
Second Q (Apr-June)	0.92 [0.91,0.94]***
Third Q (July-Sept)	0.87 [0.86,0.88]***
Fourth Q (Oct-Dec)	1.03 [1.00,1.05]*
Year (2013 Reference Group)	
2014	1.14 [1.01,1.28]*
2015	1.14 [1.02,1.27]*
2016	1.24 [1.09,1.41]***
2017	1.33 [1.18,1.49]***
2018	1.37 [1.20,1.55]***
Post ICD9 Indicator	0.90 [0.84,0.96]**
Total Hospital Admissions per Capita	1.00 [1.00,1.00]
Constant	0.01 [0.00,0.01]***
N	1536

Note: Counties were categorized according to the number of medical dispensaries in the county at baseline (third quarter 2012) prior to the legalization of recreational cannabis (no = zero medical dispensaries; low = 1-9; high = 10+). The recreational dispensary exposure was coded using the distribution in Quarter 4 of 2016, where the 25th percentile was the first category, 50th percentile is the second, 75th is the third and 100th is the fourth. All models include quarter, year and county fixed effects, although county fixed results are suppressed from the table. *** Indicates significance at the 0.01% level, ** significance at the 1% level, * significance at the 5% level, and + indicates significance at 10% level.

© 2021 Wang GS et al. *JAMA Network Open.*
